# Social isolation, healthy lifestyle, and intrinsic capacity among older adults in China: A longitudinal study

**DOI:** 10.1016/j.jnha.2025.100583

**Published:** 2025-05-22

**Authors:** Xiao Yue, Quan Yuan, Rong Zhou, Mei Wang

**Affiliations:** a Department of Geriatric Medicine, Tongji Hospital, Tongji Medical College, Huazhong University of Science and Technology, Wuhan, China; b Nursing Department, Tongji Hospital, Tongji Medical College, Huazhong University of Science and Technology, WuHan, China; c School of Nursing, Tongji Medical College, Huazhong University of Science and Technology, WuHan, China

**Keywords:** Intrinsic capacity, Social isolation, Healthy lifestyle, Aging, Longitudinal study, Public health, CHARLS

## Abstract

**Objectives:**

This study aimed to investigate the independent and interactive associations of social isolation and a healthy lifestyle on intrinsic capacity (IC) among older adults in China.

**Design and setting:**

A longitudinal cohort study was conducted using data from the China Health and Retirement Longitudinal Study (CHARLS), spanning three waves (2011, 2013, and 2015) and involving adults aged 60 years and older.

**Participants:**

A total of 4495 older adults with complete data on IC, social isolation, healthy lifestyle, and relevant covariates at baseline were included in the final analysis.

**Measurements:**

IC was assessed using a composite score across five domains: locomotor, cognitive, sensory, vitality, and psychological. Social isolation was measured using an index based on living arrangements, marital status, contact with children, and social participation. A healthy lifestyle was defined based on five factors: smoking, alcohol consumption, physical activity, sleep duration, and body mass index (BMI). Multiple linear regression and linear mixed-effects models were used to examine cross-sectional and longitudinal associations. Interactive terms between social isolation and a healthy lifestyle were analyzed. Sensitivity analyses were performed by stratifying participants by age and gender.

**Results:**

At baseline, 31.0%, 62.3%, and 6.7% of participants had social isolation scores of 0, 1−2, and 3−4, respectively, while 28.5%, 37.0%, and 34.6% had healthy lifestyle scores of 0−2, 3, and 4−5, respectively. Compared to participants with no social isolation (score = 0), the IC scores decreased for participants with social isolation scores of 1−2 and 3−4 (β = −0.36, 95% confidence interval [CI]: −0.42 to −0.30) and (β = −0.65, 95% CI: −0.77 to −0.53), respectively. In contrast, adherence to a healthy lifestyle was associated with improved IC (β = 0.27, 95% CI: 0.20 to 0.34 for score 3; β = 0.54, 95% CI: 0.47 to 0.60 for score 4−5). Interactive analysis showed that a healthy lifestyle mitigated the adverse associations of social isolation on IC, but this protective effect weakened as social isolation increased. Sensitivity analyses confirmed the robustness of these findings.

**Conclusion:**

This study identified significant associations between social isolation, healthy lifestyle, and intrinsic capacity in older Chinese adults. Social isolation was negatively associated with IC, while a healthy lifestyle was positively associated with IC. The observed interaction between social isolation and healthy lifestyle highlights the importance of integrated interventions targeting both social engagement and health behaviors in supporting IC during aging.

## Graphical abstract

**Figure:**
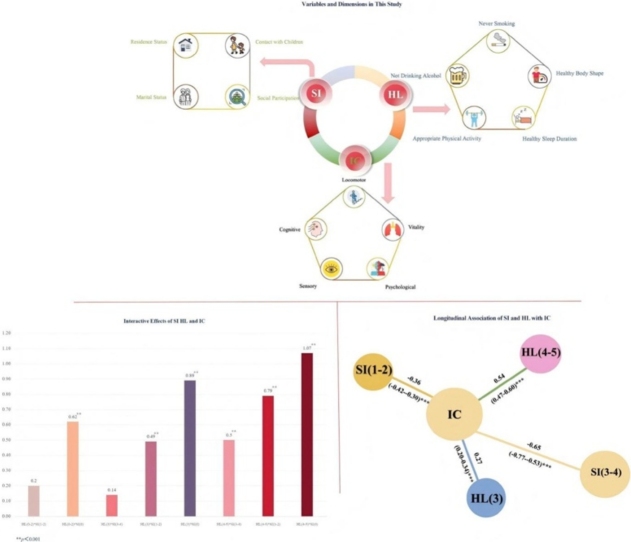

## 1 Introduction

Population aging is accelerating globally, with the number of older adults projected to exceed 1.5 billion by 2050 [1]. In China, 216.76 million individuals are aged 65 years or older, accounting for 15.4% of the population [2]. The disability rate among Chinese older adults is currently 7.0% and is expected to reach 13.68% by 2050 [3,4]. To address this challenge, the Chinese government has implemented various strategies to promote healthy aging and maintain functional ability in later life.

Intrinsic capacity (IC) refers to the composite of physical and mental capacities essential for functioning in older age, encompassing five key domains: locomotor, cognitive, vitality, sensory, and psychological, providing a comprehensive and dynamic perspective on health that reflects a shift from disease-centered aging to a more positive, function-oriented approach to healthy aging [[5], [6], [7]].

Among the modifiable social determinants of health, social isolation and healthy lifestyles have attracted growing attention [8]. Social isolation is defined as limited social interaction and a shrinking social network. Data from the National Health and Aging Trends Study (NHATS) indicate that approximately 24% of older adults are socially isolated [9], with the prevalence exceeding 30% in China [10]. Older adults often rely on community or peer support in Western cultures, while Chinese older adults are more dependent on emotional and practical support within their families, living alone or reduced interactions with family members may pose a more significant threat to functional abilities [11]. Evidence shows that social isolation is linked to some adverse health outcomes, including functional decline, cognitive impairment [12,13]. Conversely, healthy lifestyles protect physical and mental health, may help mitigate socioeconomic inequality [14,15], and are associated with slower memory decline, better cognitive function, and longer life expectancy [[16], [17], [18]].

Although previous studies have examined the associations between social isolation, healthy lifestyle, and IC, most have focused on single-variable analyses, with limited attention to their potential interactive associations. Evidence suggests that social isolation may be related to lower IC through increased psychological stress and reduced engagement in health-promoting behaviors [11,19]. Meanwhile, healthy lifestyles may help offset the negative associations of social isolation by enhancing social connections and supporting physical and mental capacities [20,21]. Therefore, this study aimed to examine the independent and interactive associations of social isolation and healthy lifestyle with IC among older adults in China, using nationally representative longitudinal data from CHARLS.

## 2 Methods

**Fig. 1:**
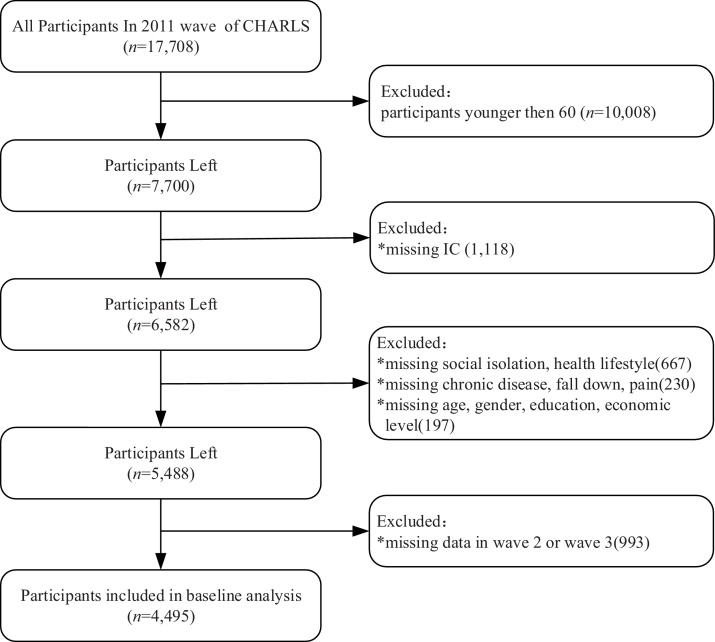
Participant selection process.

The CHARLS [22] is a nationwide longitudinal survey targeting Chinese adults aged 45 and above. It was conducted in 2011, 2013, 2015, 2018, and 2020, spanning 150 counties and 450 communities across 28 provinces. All participants provided informed consent before participating. In this study, we used data from the first three waves (2011, 2013, and 2015), as the 2018 and 2020 waves lacked key physical measurements for IC, particularly in the locomotor and vitality domains (e.g., SPPB, grip strength, lung function). These data were structurally missing and could not be imputed, and thus were excluded from analysis. Participants included in the study were aged 60 years or older and had complete data on IC. Excluding respondents missing social isolation, healthy lifestyle, and other covariates at baseline and during follow-up, and the final sample contained 4495 older adults. Fig. 1 shows the flowchart of participant screening.

### 2.1 Measurements

#### 2.1.1 Outcome variable: intrinsic capacity

We defined the scoring criteria for IC based on the ICOPE guidelines [23], the systematic review of Lopez-Ortiz et al. [24], and the cohort studies of Zhao et al. [25], Luo et al. [26], with a score of 0−10. The overall measure of IC consisted of 5 dimensions: locomotor, cognitive, sensory, vitality, and psychological. (1) Locomotor dimension: locomotor function measured with reference to the SPBB scale, which includes assessment of balance, walking speed, and 5 chair-stand tests. (2) Cognitive dimensions: cognitive function in CHARLS was assessed using the Telephone Interview of Cognitive Status scale, which was adapted from the Health and Retirement Study in the USA, which consisted of two components, intelligence level (numerical ability, temporal orientation, and visuospatial ability) and situational memory (immediate and delayed memory). (3) Sensory dimension: Sensory status assessed by self-reported hearing and visual impairment. (4) Vitality dimension: the vitality dimension was assessed by two indicators: lung capacity and grip strength. (5) Psychological dimension: psychological status was determined by scores on the Center for Epidemiologic Studies Depression Scale (CESD-10). Each IC domain was categorized into 3 categories, severely impaired, mildly impaired, and optimal, and was assigned a numerical score ranging from 0 to 2. The IC composite score was the sum of the scores of the 5 domains ranging from 0 to 10, with higher scores indicating better IC. A bifactor validated factor analysis constructed using Mplus software showed a good model fit: χ² = 1075.944, df = 123, RMSEA = 0.037, CFI = 0.946, TLI = 0.926. More details on the measurement of each domain of IC as well as the Mplus codes are described in the Supplementary material (Appendix B).

#### 2.1.2 Independent variables

##### 2.1.2.1 Social isolation

According to previous studies [27,28], the social isolation index was created based on social networks and social engagement, consists of four dichotomous indicators, residence status (1 point for those living alone), marital status (1 point for those currently unmarried, including separated, divorced, widowed and unmarried), contact with children (1 point for those who have face-to-face contact with their children or who are in contact with their children by phone/e-mail less than once a week) and social participation (1 point for those who participate in any social activity less than once a month). Social isolation is the sum of these dichotomous indicators and ranges from 0 to 4, with higher scores indicating greater social isolation. Based on the total score, social isolation is categorized into three groups: subgroup 0, subgroup 1−2, and subgroup 3−4.

##### 2.1.2.2 Healthy lifestyle

Based on the studies of Shao et al., Liu et al., and Guo et al. [[29], [30], [31]], we defined a healthy lifestyle as never smoking, not drinking alcohol, appropriate physical activity, healthy sleep duration, and a healthy body shape, with a score of 1 for each criterion met, and a total score of 0−5. Non-smoking includes those who do not smoke now as well as those who have quit smoking; non-drinking is defined as those who have consumed alcohol less than once a week in the past 1 year; and appropriate physical activity is defined as at least 3 times a week, 30 min of intense physical activity or moderate physical activity each time; healthy sleep duration was defined as greater than or equal to 7 h of sleep per night; body size was judged based on body mass index (BMI = weight (kg)/height^2^ (m^2^)), and a healthy body size was defined as 18.5 ≤ BMI < 24 kg/m^2^ when the age was less than 80 years old, and 22.0 kg/m^2^ ≤ BMI < 26.9 kg/m^2^ when the age was greater than or equal to 80 years old [32]. Based on the total score, healthy lifestyles were classified into 3 categories: subgroups 0−2, subgroup 3, and subgroups 4−5 subgroups.

#### 2.1.3 Other covariates

Covariates included sociodemographic, socioeconomic and health-related factors that may be associated with IC [31,[33], [34], [35], [36], [37]]. Sociodemographic factors included age, gender, education level (illiteracy, primary school, middle school, high school and above). Economic status was assessed based on per household consumption expenditure and categorized into three equal tertiles (low, medium, and high). Health-related factors included pain, chronic diseases, and falls. A more detailed description of health-related covariates is provided in the Supplementary material.

### 2.2 Statistical analysis

Descriptive statistics were used to summarize baseline characteristics. Continuous variables were presented as means with standard deviations (SD), and categorical variables as frequencies and percentages. Baseline differences across categories of social isolation and healthy lifestyle were analyzed using chi-square tests for categorical variables and Kruskal–Wallis tests for continuous variables. IC was the outcome variable in both cross-sectional and longitudinal analyses. Cross-sectional associations between social isolation, healthy lifestyle, and IC were examined using multiple linear regression models. For the longitudinal analyses, IC, all exposure variables, and covariates were repeatedly measured across three waves (2011, 2013, and 2015), and were modeled using a linear mixed-effects model with IC as a continuous dependent variable. Fixed effects included social isolation, healthy lifestyle, their interaction term, and relevant covariates, while random effects included time and a subject-specific random intercept to account for intra-individual variability. In the modeling process, social isolation, healthy lifestyle, and covariates such as economic level and health-related factors (e.g., chronic disease, pain, and falls) were treated as time-varying variables. Results were presented as regression coefficients (β) with 95% CI. A series of stepwise models were constructed as follows, Model 1 (included only social isolation variables), Model 2 (included only healthy lifestyle variables), Model 3 (included both social isolation and healthy lifestyle variables), Model 4 (Model 3 with additional adjustment for sociodemographic covariates, including age, gender, education level, and economic status), Model 5 (Model 4 further adjusted for health-related covariates, including pain, falls, and chronic disease). Covariates were selected based on prior literature, biological plausibility, or statistical significance (*p* <  0.05). Interaction effects between social isolation and healthy lifestyle were assessed in fully adjusted models. Sensitivity analyses included stratification by gender and age. Missing data were handled using multiple imputation. All statistical analyses were performed using the Windows versions of Stata version 18.0 (StataCorp LLC, College Station, TX) and IBM SPSS Statistics version 26.0 (IBM Corp., Armonk, NY). A two-sided p-value < 0.05 was considered statistically significant.

## 3 Results

### 3.1 Participant flow and sample profile

We included 4742 participants aged 60 and older in the final analysis, with 3853 followed up in 2013 and 3401 in 2015 (Fig. 1), the cumulative attrition rate over the 4-year follow-up period was approximately 19.8%. A total of 2087 participants were excluded due to missing data on key variables (e.g., social isonlation, health lifestyle, pain, etc.). Univariate analysis revealed that participants with incomplete data were older, and less educated (*p* < 0.05), No significant differences were observed in gender, economic level, pain, chronic disease, or other characteristics (Supplementary Table S1). Characteristics of the included participants and their IC scores at baseline are presented in Table 1. The median age was 66 years (interquartile range [IQR], 62–71), and 51.5% were male. Overall, 53.8% of participants were illiterate, 65.3% reported no pain, and 54.5% had multimorbidity. Regarding economic level, 38.6% of participants were classified as low, 31.5% as medium, and 29.9% as high. Social isolation was observed in 31.0%, 62.3%, and 6.7% of participants in the score subgroups of 0, 1−2, and 3−4, respectively. Additionally, 28.5% had healthy lifestyle scores of 0−2, 37.0% scored 3, and 34.6% scored 4−5.

**Table 1:** Characteristics of the older participants and IC at baseline.

Characteristic	*N* (%)	IC	*p*
Age, years, Median (IQR)	66.00(62.00−71.00)	6.67 ± 1.76	<0.001
Gender *N* (%): Female	2182(48.5)	6.34 ± 1.83	<0.001
Male	2313(51.5)	6.98 ± 1.64	
Education *N* (%): Illiteracy	2417(53.8)	6.15 ± 1.79	<0.001
Primary school	1190(26.5)	7.10 ± 1.56	
Middle School	588(13.1)	7.47 ± 1.42	
High school and above	300(6.7)	7.56 ± 1.41	
Economic level *N* (%): Low	1733(38.6)	6.40 ± 1.82	<0.001
Medium	1418(31.5)	6.67 ± 1.75	
High	1344(29.9)	7.03 ± 1.63	
Pain *N* (%): No pain	2936(65.3)	7.09 ± 1.62	<0.001
Single-site pain	317(7.1)	6.45 ± 1.79	
Multisite pain	1242(27.6)	5.75 ± 1.71	
Chronic disease *N* (%): None	677(15.1)	7.03 ± 1.62	<0.001
Singe	1366(30.4)	6.90 ± 1.70	
Multimorbidity	2452(54.5)	6.45 ± 1.80	
Fall in the past 2 years *N* (%): Yes	849(18.9)	6.82 ± 1.70	<0.001
No	3646(81.1)	6.02 ± 1.86	
Social Isolation(scores): 0	1392(31.0)	7.17 ± 1.61	<0.001
1−2	2800(62.3)	6.51 ± 1.76	
3−4	303(6.7)	5.94 ± 1.84	
Health Lifestyle(scores): 0−2	1280(28.5)	6.35 ± 1.73	<0.001
3	1661(37)	6.69 ± 1.77	
4−5	1554(34.6)	6.92 ± 1.73	

Note: IQR = interquartile range; IC = intrinsic capacity.

Participants had a mean IC score of 6.67 ± 1.76, which was lower in those with higher social isolation and higher in those with better health behaviors. Baseline characteristics and IC levels stratified by social isolation and healthy lifestyle scores are presented in Supplementary Table S2. Significant differences in social isolation and healthy lifestyle scores were observed across age, gender, education, pain, and other baseline characteristics.

### 3.2 The cross-sectional study

Supplementary Table S3 shows the cross-sectional associations of social isolation and healthy lifestyle scores with IC among older adults. We found higher social isolation was associated with lower IC (B: −0.39 to −0.60; *p* <  0.001), while healthier lifestyle scores were positively associated with IC (B: 0.35 to 0.58; *p* <  0.001). Among covariates, older age, female gender, lower education, lower economic status, pain, multimorbidity, and history of falls were linked to lower IC.

### 3.3 The longitudinal study

The results in Table 2 show the longitudinal relationship between social isolation, healthy lifestyle, and IC. All models showed consistent results: higher social isolation reduced IC, and a healthier lifestyle improved it (A detailed description of the results of Model 1- Model 4 is provided in the Supplementary Material). About Model 5, which was fully adjusted for gender, age, education, pain, fall down, and chronic disease, the IC scores decreased for participants with social isolation scores of 1−2 and 3−4 (β = −0.36, 95% CI: −0.42 to −0.30) and (β = −0.65, 95% CI: −0.77 to −0.53), respectively, and the IC increased for healthy lifestyle scores of 3 and 4−5 for participants (β = 0.27, 95% CI: 0.20 to 0.34) and (β = 0.54, 95% CI: 0.47 to 0.60), respectively.

**Table 2:** Longitudinal association of social isolation and health lifestyle with IC among older adults.

Characteristics	Model 1	Model 2	Model 3	Model 4	Model 5
	β(95%CI) p-value	β(95%CI) p-value	β(95%CI) p-value	β(95%CI) p-value	β(95%CI) p-value
Social Isolation(scores) (ref: 0)					
01-Feb	−0.65 (−0.71 to −0.58)***	NA	−0.65 (−0.72 to −0.59)***	−0.40 (−0.46 to −0.33)***	−0.36 (−0.42 to −0.30)***
03-Apr	−1.30 (−1.43 to −1.17)***	NA	−1.31 (−1.44 to −1.17)***	−0.69 (−0.81 to −0.57)***	−0.65 (−0.77 to −0.53)***
Health Lifestyle(scores) (ref: 0−2)					
3	NA	0.26 (0.18−0.34)***	0.27 (0.20−0.35)***	0.33 (0.26−0.40)***	0.27 (0.20−0.34)***
04-May	NA	0.58 (0.50−0.66)***	0.59 (0.51−0.67)***	0.68 (0.61−0.75)***	0.54 (0.47−0.60)***
AIC	45,947.8	46,277.9	45,732.3	43,757.5	42,567.2
BIC	45,962.5	46,292.6	45,747.1	43,772.2	42,582.0

Note: IC = intrinsic capacity; NA = not applicable.

Boldface indicates statistical significance (*** *p* < 0.001).

Model 1, 2, 3 was unadjusted.

Model 1: included social isolation variables.

Model 2: included health lifestyle variables.

Model 3: included both social isolation and health lifestyle variables.

Model 4: Model 3 was adjusted for age, gender, education, economic level.

Model 5: Model 3 was adjusted for age, gender, education, economic level, pain, fall down, and chronic disease.

**Fig. 2:**
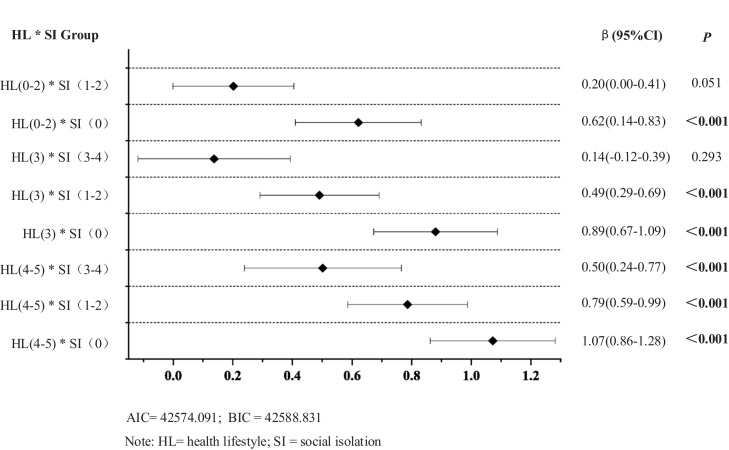
Interactive effects of social isolation and healthy lifestyle on intrinsic capacity.

Fig. 2 presents the interactive associations of social isolation and healthy lifestyle with IC, using HL (0−2) * SI (3−4) as the reference group. Among older adults with high adherence to a healthy lifestyle (score 4−5), the association with higher IC was strongest in the absence of social isolation (score = 0: β = 1.07, 95% CI: 0.86–1.28), and progressively weakened as social isolation increased (score = 1−2: β = 0.79, 95% CI: 0.59 to 0.99; score = 3−4: β = 0.50, 95% CI: 0.24 to 0.77). For individuals with moderate adherence to a healthy lifestyle (score = 3), a similar but weaker trend was observed. The association with IC remained statistically significant when social isolation was low or moderate (score = 0: β = 0.89, 95% CI: 0.67–1.09; score = 1−2: β = 0.49, 95% CI: 0.29 to 0.69), but was no longer significant at the highest level of social isolation (score = 3−4: β = –0.14, 95% CI: –0.12 to 0.39, *p* =  0.293). These findings suggest that moderate adherence to a healthy lifestyle may be insufficient to counteract the association between severe social isolation and lower IC. In contrast, for individuals with low adherence to a healthy lifestyle (score 0−2), no significant interaction was observed at higher levels of social isolation(score = 1−2: β = –0.20, 95% CI: 0.00 to 0.41, *p* =  0.051).

Supplementary Table S3 presents the longitudinal associations between social isolation, healthy lifestyle, and individual IC domains. Higher levels of social isolation were associated with lower scores in cognitive and psychological capacities over time, whereas higher scores of healthy lifestyle was linked to better performance in locomotion and psychological domains.

Table 3 shows the results of the sensitivity analyses. The associations of social isolation and healthy lifestyle with IC remained consistent across age and gender groups, confirming the robustness of the findings from the primary models (Table 2).

**Table 3:** Longitudinal association of Social Isolation, Health Lifestyle and IC by subgroups of age, sex.

Characteristics	Age(years)		Gender	
	Model 1	Model 2	Model 3	Model 4
	<75	≥75	Male	Female
	β(95%CI) p-value	β(95%CI) p-value	β(95%CI) p-value	β(95%CI) p-value
Social Isolation(scores) (ref: 0)				
1−2	−0.38 (−0.44 to −0.31)***	−0.47 (−0.63 to −0.31)***	−0.32 (−0.40 to −0.25)***	−0.42 (−0.51 to −0.32)***
3−4	−0.72 (−0.86 to −0.58)***	−0.79 (−1.03 to −0.56)***	−0.51 (−0.68 to −0.34)***	−0.76 (−0.92 to −0.59)***
Health Lifestyle (scores) (ref: 0−2)				
3	0.27 (0.19−0.34)***	0.33 (0.17−0.49)***	0.25 (0.16−0.33)***	0.30 (0.19−0.40)***
4−5	0.57 (0.49−0.64)***	0.44 (0.26−0.61)***	0.52 (0.43−0.61)***	0.56 (0.45−0.67)***
AIC	34,876.958	7897.572	21,601.704	20,919.971
BIC	34,891.304	7908.858	21,615.136	20,933.238

Note: IC = intrinsic capacity.

Boldface indicates statistical significance (*** *p* < 0.001).

Model 1, 2 were adjusted for gender, education, economic level, pain, fall down, and chronic disease.

Model 3, 4 were adjusted for age, education, economic level, pain, fall down, and chronic disease.

## 4 Discussion

This study provides comprehensive evidence of the independent and interactive associations of social isolation and healthy lifestyle on IC among older adults. Cross-sectional analyses revealed that social isolation was negatively associated with IC, with greater isolation corresponding to significantly lower IC scores, while a healthy lifestyle was positively associated with IC. Longitudinal analyses further supported these findings, showing that higher levels of social isolation were consistently associated with declines in IC over time, whereas healthier lifestyles were linked to improvements in IC. Importantly, the interaction analysis revealed that the association between healthy lifestyle and IC varied by level of social isolation. Specifically, healthier lifestyles were associated with higher IC particularly when social isolation was low, while this association appeared to weaken as social isolation increased. Sensitivity analyses confirmed the consistency of these findings across age and gender groups, emphasizing the relevance of social and behavioral factors in supporting IC in aging populations.

Our study found that older adults with higher social isolation scores had significantly lower IC scores. This finding aligns with previous studies and supports the observed associations between social isolation and reduced functional health in older adults. Studies by Shen et al. and Jang et al. have noted that social isolation not only leads to loneliness, but is also associated with declining cognitive functioning and deteriorating health [38,39]. Jiang et al. also found that older adults living alone were at higher risk of reduced IC (95% CI: 1.13–3.22) [40]. Several mechanisms may explain these associations. First, individuals with higher levels of social isolation have fewer social support networks and resources to bring to bear in the face of health problems, and thus those with a sense of loneliness are more likely to exhibit fewer health-promoting behaviors, which may contribute to a decline in multiple functional domains [41,42]. Second, social isolation has been associated with chronic stress, which may impair physiological regulatory systems, including brain function, thereby affecting both cognitive and physical capacities [43]. Additionally, social isolation may reduce the opportunities for older adults to engage in cognitive and physical activities, and increase the risk of motor cognitive syndrome, thus further decreasing their IC [44]. These findings suggest that strengthening social networks and facilitating opportunities for meaningful interaction may be beneficial in supporting IC among older adults.

In contrast to the negative associations observed with social isolation, maintaining a healthy lifestyle was positively associated with higher IC scores. Our findings suggest that even in the context of social isolation, a healthy lifestyle may be linked to better IC outcomes, consistent with previous research by Muneera et al. and Jiang et al., which reported positive associations between healthy lifestyle behaviors and IC in older adults [40,45]. A scoping review further identified unhealthy behaviors (such as smoking, excessive alcohol consumption, and physical inactivity) as potential risk factors for lower IC [46]. Harmful compounds in cigarettes are not only cognitively damaging, but have also been linked to eye diseases such as cataracts and glaucoma [47,48]. Angelsen et al. used 12,361 participants in the HUNT study to confirm associations between IC and social interactions, fruit and vegetable intake, and levels of physical activity, which is consistent with the findings of this study [49]. Previous research has also indicated that physical activity is associated with better functional performance, cognitive capacity, and psychological well-being among older adults, and may reduce unnecessary dependence on healthcare services [50,51]. Zhao et al. reported that participants with hearing loss combined with an unhealthy lifestyle exhibited approximately twice the risk of cognitive impairment compared to participants with normal hearing and a healthy lifestyle, however, adherence to a healthy lifestyle in patients with hearing loss significantly reduced the cognitive impairment risk [52]. In addition, healthy lifestyle stimulate positive emotions and lead to better subjective well-being among Chinese older adults [15]. These findings support the value of promoting healthy lifestyle behaviors in later life, not only for enhancing individual well-being but also as a potential focus for public health strategies aimed at supporting intrinsic capacity in aging populations.

Our study analyzed the interaction between healthy lifestyle and social isolation in relation to IC and found that the positive association between a healthy lifestyle and IC became weaker as the level of social isolation increased. This indicates that the association between health behaviors and IC may vary depending on the degree of social isolation, and may be less pronounced in individuals experiencing higher levels of isolation. In domain-specific analyses, we found that the associations of social isolation and healthy lifestyle with IC were not uniform across its five dimensions. Social isolation had the strongest associations with declines in cognitive and psychological capacities, which may be explained by the emotional and cognitive vulnerabilities associated with limited social engagement [41,43]. In contrast, a healthy lifestyle was most positively associated with locomotor and psychological functioning, potentially reflecting the role of health lifestyle in maintaining mobility and mental well-being [53,54]. These findings highlight the need for further research into the dynamic interplay between behavioral and social factors over time, including potential reciprocal and mediating pathways through which lifestyle behaviors and social isolation may be interrelated and jointly associated with IC in later life. Additionally, it is important to account for domain-specific patterns when developing strategies to support or maintain IC in aging populations.

This study also explored the associations of demographic factors such as age, gender, education, and economic level on IC. The results showed that increasing age was associated with decreasing IC, women tended to have lower IC than men, and lower levels of education and economic status were linked to lower IC. These findings are consistent with those reported by Huang et al. and Zhao et al. [25,55]. The relatively high proportion of illiteracy in our sample reflects the demographic characteristics of the older Chinese population, particularly those born before the 1950s, who had limited access to formal education [56]. Future research should explore tailored late-life educational interventions aimed at improving health literacy and cognitive reserve, which may potentially enhance IC among older adults with limited formal educational backgrounds. In addition, health-related factors such as multisite pain, chronic diseases, and falls were also associated with lower IC, aligning with previous studies on healthy aging [34,46,57]. These results suggest that assessments of IC in older adults may benefit from taking into account pain, chronic conditions, and fall history. Consideration of these factors could help inform more individualized approaches to health monitoring and support in this population.

## 5 Limitations and strengths

While this study offers valuable insights, several limitations should be acknowledged. First, the geographic and cultural specificity of the sample may limit the generalizability of the findings to broader populations, highlighting the need for replication in more diverse regional and cultural contexts. Second, the five-year follow-up period may not fully capture longer-term trajectories or dynamic changes in IC. Future studies with extended follow-up durations would be valuable for examining these patterns over time. Third, although we employed longitudinal modeling and adjusted for baseline differences in IC, the associations between IC, social isolation, and healthy lifestyle behaviors may still be subject to bidirectional influences. Individuals with lower IC at baseline may be more likely to experience social isolation or engage in unhealthy behaviors, which in turn may further reduce IC over time. While our use of linear mixed-effects models and sensitivity analyses helps mitigate this concern, causal relationships cannot be fully established in observational studies. Future research using cross-lagged models or experimental designs is needed to further disentangle these complex relationships. Nevertheless, the study has several strengths. It utilizes a nationally representative cohort, incorporates repeated IC measurements across three waves, and integrates both social and behavioral factors within a single framework to better understand functional aging. Furthermore, modeling key exposures such as social isolation and healthy lifestyle as time-varying variables across three survey waves further strengthens the temporal validity of our findings.

## 6 Conclusions and implications

This study highlights the significant and interconnected roles of social isolation and healthy lifestyle in shaping IC among older adults. The findings demonstrate that social isolation negatively impacts IC, while adherence to a healthy lifestyle positively contributes to maintaining or improving IC. Furthermore, the interaction analysis reveals that a healthy lifestyle can partially mitigate the detrimental effects of social isolation, although its protective effect diminishes as the severity of social isolation increases. These results underscore the compounded risks faced by older adults experiencing both high social isolation and suboptimal health behaviors.

## CRediT authorship contribution statement

We confirm that all authors meet the authorship criteria as outlined in the Uniform Requirements for Manuscripts Submitted to Biomedical Journals, and all authors have reviewed and approved the submission of this manuscript.

Note: Xiao Yue, Quan Yuan contributed equally to this work and share the first authorship

• Xiao Yue: Conceptualization, Data curation, Formal analysis, Methodology, Software, Visualization, Writing - original draft.
• Quan Yuan: Conceptualization, Data curation, Formal analysis, Funding acquisition, Methodology, Software, Writing - original draft.
• Rong Zhou: Conceptualization, Methodology, Software, Visualization, Writing - review & editing.
• Mei Wang: Conceptualization, Data curation, Formal analysis, Investigation; Methodology, Project administration, Supervision, Writing - review & editing.

## Ethical approval

The ethics application for collecting data on human subjects in CHARLS was approved by the Biomedical Ethics Review Committee of Peking University (IRB00001052-11015), and all CHARLS participants provided written informed consent.

Clinical trial number: not applicable.

## Brief summary

Our study on older adults in China demonstrates that social isolation is significantly associated with a decline in intrinsic capacity (IC), while maintaining three or more healthy lifestyles has a protective effect. These findings suggest that interventions aimed at reducing social isolation and promoting healthier living can be vital in preserving IC in older adults.

## Declaration of Generative AI and AI-assisted technologies in the writing process

During the preparation of this work, the authors used ChatGPT, an AI language model provided by OpenAI, to improve readability and enhance the clarity of the manuscript. After using this tool, the authors carefully reviewed and edited the content to ensure accuracy and take full responsibility for the content of this publication.

## Funding declaration

This work was supported by 10.13039/501100003819Natural Science Foundation of Hubei Province (Grant/Award Number: 2023AFB763); Natural Science Foundation of Hubei Province ( Grant/Award Number: 2024AFB903); Research Fund project of Tongji Hospital Affiliated to Tongji Medical College, 10.13039/501100003397Huazhong University of Science and Technology, Grant/Award Number: 2024D07.

## Availability of data and materials

The data sets analyzed in this study can be accessed in the China Health and Retirement Longitudinal Study (CHARLS; https://charls.pku.edu.cn/).

## Declaration of competing interest

All authors declare no conflict of interest.

## References

[1] United Nations Department of Economic and Social Affairs (2019). World population ageing 2019: highlights.

[2] Ministry of Civil Affairs of the People’s Republic of China, Department of Ageing Health (2023). The 2023 bulletin on the development of national undertakings for the older adults.

[3] Yu P., Gao C., Lei P., Liu X., Zhang C., Wang J. (2019). Chinese expert consensus on core information for disability prevention in the elderly. Chin J Geriatr.

[4] Wang J., Li T. (2020). The age model of old age disability and the prediction of future disability population in China. Popul J.

[5] Venkatapuram S., Amuthavalli Thiyagarajan J. (2023). The capability approach and the WHO healthy ageing framework (for the UN decade of healthy ageing). Age Ageing.

[6] George P.P., Lun P., Ong S.P., Lim W.S. (2021). A Rapid review of the measurement of intrinsic capacity in older adults. J Nutr Health Aging.

[7] Sánchez-Sánchez J.L., Lu W.H., Gallardo-Gómez D., Del Pozo Cruz B., de Souto Barreto P., Lucia A. (2024). Association of intrinsic capacity with functional decline and mortality in older adults: a systematic review and meta-analysis of longitudinal studies. Lancet Healthy Longev.

[8] Chunara R., Gjonaj J., Immaculate E., Wanga I., Alaro J., Scott-Sheldon L.A.J. (2024). Social determinants of health: the need for data science methods and capacity. Lancet Digit Health.

[9] Guo L., An L., Luo F., Yu B. (2021). Social isolation, loneliness and functional disability in Chinese older women and men: a longitudinal study. Age Ageing.

[10] Lin Y., Zhu T., Zhang X., Zeng Z. (2024). Trends in the prevalence of social isolation among middle and older adults in China from 2011 to 2018: the China Health and Retirement Longitudinal Study. BMC Public Health.

[11] Su H., Xu L., Yu H., Zhou Y., Li Y. (2023). Social isolation and intrinsic capacity among left-behind older adults in rural China: the chain mediating effect of perceived stress and health-promoting behavior. Front Public Health.

[12] Shankar A., McMunn A., Demakakos P., Hamer M., Steptoe A. (2017). Social isolation and loneliness: prospective associations with functional status in older adults. Health Psychol.

[13] Evans I.E.M., Martyr A., Collins R., Brayne C., Clare L. (2019). Social isolation and cognitive function in later life: a systematic review and meta-analysis. J Alzheimers Dis.

[14] Ren Z., Du Y., Lian X., Sun J., Zheng X., Liu J. (2023). The dilution effects of healthy lifestyles on the risk of depressive symptoms attributed to life-course disadvantages among Chinese middle-aged and older adults. J Affect Disord.

[15] Zhang L., Bi X., Ding Z. (2021). Health lifestyles and Chinese oldest-old’s subjective well-being-evidence from a latent class analysis. BMC Geriatr.

[16] Jia J., Zhao T., Liu Z., Liang Y., Li F., Li Y. (2023). Association between healthy lifestyle and memory decline in older adults: 10 year, population based, prospective cohort study. BMJ.

[17] Dhana K., Agarwal P., James B.D., Leurgans S.E., Rajan K.B., Aggarwal N.T. (2024). Healthy lifestyle and cognition in older adults with Common neuropathologies of dementia. JAMA Neurol.

[18] Wang J., Chen C., Zhou J., Ye L., Li Y., Xu L. (2023). Healthy lifestyle in late-life, longevity genes, and life expectancy among older adults: a 20-year, population-based, prospective cohort study. Lancet Healthy Longev.

[19] Guo W., Meng L., Han J., Yang B., Sun J., Guo Y. (2024). Intrinsic capacity and its association with predictors among Chinese empty nest older adults in communities: a latent class analysis. BMC Geriatr.

[20] Paquet C., Whitehead J., Shah R., Adams A.M., Dooley D., Spreng R.N. (2023). Social prescription interventions addressing social isolation and loneliness in older adults: meta-review integrating on-the-Ground resources. J Med Internet Res.

[21] Luo M., Ding D., Bauman A., Negin J., Phongsavan P. (2020). Social engagement pattern, health behaviors and subjective well-being of older adults: an international perspective using WHO-SAGE survey data. BMC Public Health.

[22] Zhao Y., Hu Y., Smith J.P., Strauss J., Yang G. (2014). Cohort profile: the China Health and Retirement Longitudinal Study (CHARLS). Int J Epidemiol.

[23] World Health Organization (2024). Integrated care for older people (ICOPE): guidance for person-centred assessment and pathways in primary care.

[24] López-Ortiz S., Lista S., Peñín-Grandes S., Pinto-Fraga J., Valenzuela P.L., Nisticò R. (2022). Defining and assessing intrinsic capacity in older people: a systematic review and a proposed scoring system. Ageing Res Rev.

[25] Zhao B., Liu Z., Fu Y., Zhang H., Wu J., Lai C. (2024). Social determinants of intrinsic capacity: a national cohort study. Am J Prev Med.

[26] Luo Y., Pan X., Zhang Z. (2019). Productive activities and cognitive decline among older adults in China: evidence from the China Health and Retirement Longitudinal Study. Soc Sci Med.

[27] Lin L., Cao B., Chen W., Li J., Zhang Y., Guo V.Y. (2022). Association of adverse childhood experiences and social isolation with later-life cognitive function among adults in China. JAMA Netw Open.

[28] Luo F., Guo L., Thapa A., Yu B. (2021). Social isolation and depression onset among middle-aged and older adults in China: moderating effects of education and gender differences. J Affect Disord.

[29] Liu Y., Du Q., Jiang Y. (2023). Detection rate of decreased intrinsic capacity of older adults: a systematic review and meta-analysis. Aging Clin Exp Res.

[30] Shao J., Wang X., Zou P., Song P., Chen D., Zhang H. (2021). Associating modifiable lifestyle factors with multimorbidity in community dwelling individuals from mainland China. Eur J Cardiovasc Nurs.

[31] Guo C., Liu Z., Fan H., Wang H., Zhang X., Fan C. (2023). Associations of healthy lifestyle and three latent socioeconomic status patterns with physical multimorbidity among middle-aged and older adults in China. Prev Med.

[32] Shi X., Chinese Nutrition Society (2023). Appropriate range of body mass index and body weight management guidelines for Chinese oldest old (T/CNSS 021-2023). Chin J Epidemiol.

[33] Zhao Y., Chen Y., Xiao L.D., Liu Q., Nan J., Li X. (2024). Intrinsic capacity trajectories, predictors and associations with care dependence in community-dwelling older adults: a social determinant of health perspective. Geriatr Nurs.

[34] Meng L.C., Chuang H.M., Lu W.H., Lee W.J., Liang C.K., Loh C.H. (2023). Multi-trajectories of intrinsic capacity decline and their impact on age-related outcomes: a 20-year national longitudinal cohort study. Aging Dis.

[35] Meng L.C., Hsiao F.Y., Huang S.T., Lu W.H., Peng L.N., Chen L.K. (2022). Intrinsic capacity impairment patterns and their associations with unfavorable medication utilization: a nationwide population-based study of 37,993 Community-dwelling older adults. J Nutr Health Aging.

[36] Zhao Y., Atun R., Oldenburg B., McPake B., Tang S., Mercer S.W. (2020). Physical multimorbidity, health service use, and catastrophic health expenditure by socioeconomic groups in China: an analysis of population-based panel data. Lancet Glob Health.

[37] (2017). Integrated care for older people: guidelines on community-level interventions to manage declines in intrinsic capacity.

[38] Shen C., Rolls E.T., Cheng W., Kang J., Dong G., Xie C. (2022). Associations of social isolation and loneliness with later dementia. Neurology.

[39] Jang Y., Choi E.Y., Park N.S., Chiriboga D.A., Duan L., Kim M.T. (2021). Cognitive health risks posed by social isolation and loneliness in older Korean Americans. BMC Geriatr.

[40] Jiang X., Chen F., Yang X., Yang M., Zhang X., Ma X. (2023). Effects of personal and health characteristics on the intrinsic capacity of older adults in the community: a cross-sectional study using the healthy aging framework. BMC Geriatr.

[41] Shah S.J., Fang M.C., Wannier S.R., Steinman M.A., Covinsky K.E. (2022). Association of social support with functional outcomes in older adults who live alone. JAMA Intern Med.

[42] Shamlou R., Nikpeyma N., Pashaeipour S., Sahebi L., Mehrgou Z. (2021). Relationship of loneliness and social isolation with self-care ability among older adults. J Psychosoc Nurs Ment Health Serv.

[43] Cacioppo J.T., Cacioppo S., Capitanio J.P., Cole S.W. (2015). The neuroendocrinology of social isolation. Annu Rev Psychol.

[44] Zhou C., Wu F. (2023). Social isolation, loneliness, and motoric cognitive risk syndrome among older adults in China: a longitudinal study. Int J Geriatr Psychiatry.

[45] Muneera K., Muhammad T., Althaf S. (2022). Socio-demographic and lifestyle factors associated with intrinsic capacity among older adults: evidence from India. BMC Geriatr.

[46] Wei X., Chen Y., Qin J., Yang Y., Yang T., Yan F. (2024). Factors associated with the intrinsic capacity in older adults: a scoping review. J Clin Nurs.

[47] Morley J.E. (2018). An overview of cognitive impairment. Clin Geriatr Med.

[48] Hu J.Y., Yan L., Chen Y.D., Du XH Li T.T., Liu D.A. (2017). Population-based survey of prevalence, causes, and risk factors for blindness and visual impairment in an aging Chinese metropolitan population. Int J Ophthalmol.

[49] Angelsen A., Nakrem S., Zotcheva E., Strand B.H., Strand L.B. (2024). Health-promoting behaviors in older adulthood and intrinsic capacity 10 years later: the HUNT study. BMC Public Health.

[50] Pahor M., Guralnik J.M., Ambrosius W.T., Blair S., Bonds D.E., Church T.S. (2014). Effect of structured physical activity on prevention of major mobility disability in older adults: the LIFE study randomized clinical trial. JAMA.

[51] Bigarella L.G., Ballotin V.R., Mazurkiewicz L.F., Ballardin A.C., Rech D.L., Bigarella R.L. (2022). Exercise for depression and depressive symptoms in older adults: an umbrella review of systematic reviews and Meta-analyses. Aging Ment Health.

[52] Zhao F., Wang Z., Wu Z., Wang X., Li Y., Gao Y. (2024). Joint association of combined healthy lifestyle factors and hearing loss with cognitive impairment in China. J Gerontol A Biol Sci Med Sci.

[53] Li N., Wang N., Xu Y., Si L., Yin Y., Feng H. (2025). The impacts of a mHealth platform-enabled lifestyle-integrated multicomponent exercise program on reversing pre-frailty in community-dwelling older adults: a randomized controlled trial. Int J Nurs Stud.

[54] Cao Z., Min J., Xiang Y.T., Wang X., Xu C. (2024). Healthy lifestyle and the risk of depression recurrence requiring hospitalisation and mortality among adults with pre-existing depression: a prospective cohort study. BMJ Ment Health.

[55] Huang Z.T., Lai E.T.C., Luo Y., Woo J. (2024). Social determinants of intrinsic capacity: a systematic review of observational studies. Ageing Res Rev.

[56] Wang J., Zhang G., Tian L. (2024). Reconstruction of China’s population age and education structure from 2000 to 2020—based on the data from the 5th to 7th national population censuses in China. China Popul Dev Stud.

[57] Zhu X., Zhang X., Ding L., Tang Y., Xu A., Yang F. (2023). Associations of pain and sarcopenia with successful aging among older people in China: evidence from CHARLS. J Nutr Health Aging.

