# Supplementary material for: Social isolation, healthy lifestyle, and intrinsic capacity among older adults in China: A longitudinal study
Source: J Nutr Health Aging. 2025 May 22;29(8):100583. doi: 10.1016/j.jnha.2025.100583 (PMC12172961; doi:10.1016/j.jnha.2025.100583)
Supplement: Supplementary file 1 [file mmc1.docx]

**Supplementary Material**

**Social isolation,** **h****ealthy lifestyle, and intrinsic capacity** **among older adults in China: A longitudinal study**

[1.Scoring methods for the five domains of intrinsic capacity. 2](#_Toc2134)

[2.Evaluation methods of health-related covariates. 7](#_Toc19291)

[3.Supplementary material of Table S1 7](#_Toc7904)

[4.Supplementary material of Table S2 9](#_Toc31937)

[5. Supplementary material of Table S3 11](#_Toc3907)

[6. A detailed description of the results of Model 1- Model 4 in Table 2 12](#_Toc29439)

# 1.Scoring methods for the five domains of intrinsic capacity.

**Based on an extensive literature review, the IC score for this study was set as the sum of the scores (0-10) of the five dimensions.**

**Locomotion** **dimension**: The Simple Physical Performance Battery (SPPB) is a test recommended by the World Health Organization (WHO) for assessing exercise capacity, including balance ability in the standing position, walking speed, and the Five Times Sit-to-Stand Test.

(1)Balance ability: Three measurements were included, i.e., standing with feet in a line in front and behind, standing with feet half in front and behind, and standing with feet together. All respondents tried to perform the test of standing with feet half in front and behind. If they were able to stand for 10 seconds, the respondents were tested by standing with their feet in a line in front and behind. If the respondents could not keep standing with their feet half forward and backward for 10 seconds, they performed parallel standing with their feet. Participants who were able to stand with their feet together or stand with their feet half forward and backward for 10 seconds each received a score of 1, and those who did not hold it for 10 seconds and did not attempt to stand received a score of 0. Participants who kept their feet in a line for 10 seconds scored 2, those who kept them for 3-9.9 seconds scored 1, and those who kept them for less than 3 seconds or without attempting it scored 0.

(2) Walking speed: The participant walks twice at normal speed over a distance of 2.5 meters and is timed, using a walking aid if necessary. The faster of the two attempts was recorded in our study. The 2.5 m walking speed from the original questionnaire was converted to 4 m walking speed and walking 4 m within 4.82 seconds, 4.82-6.20 seconds, 6.21-8.70 seconds, more than 8.70 seconds and inability to complete were scored as 4, 3, 2, 1 and 0 respectively.

(3) The Five Times Sit-to-Stand Test: This was assessed by measuring the time of complete five consecutive from a seated position to a standing position. Participants sat on a standard stool equipped for the program with their arms crossed over their chests, and stood-sat down five times, as fast as they could without stopping in the middle and without bracing themselves with their hands, and the time was recorded for those who completed, with the slower completion times indicating poorer physical functioning. The time for Five Times Sit-to-Stand Test were categorized as <11.19 seconds, 11.2-13.69 seconds, 13.7-16.69 seconds, 16.7-59.9 seconds, >60 seconds, or could not be completed, and were scored as 4, 3, 2, 1, or 0, respectively.

The SPBB score was the sum of the three, and the locomotion dimension was divided into three levels, 0≤SPBB≤2, 3≤SPBB≤9, and 10≤SPBB≤12, and was assigned the score of 0, 1, 2 respectively.

**Cognitive dimension:** The cognitive function of CHARLS participants was assessed using the Telephone Interview for Cognitive Status (TICS) scale, which was adapted from the U.S. Health and Retirement Study (HRS). The scale consists of two parts: intelligence level (including numerical skills, temporal orientation, and visuospatial ability) and episodic memory (immediate and delayed recall). The intelligence level assessment includes tasks such as subtracting 7 from 100 (repeated 5 times), answering questions about the current year, month, day, week, and season, and drawing a figure (two overlapping pentagons), with a score range of 0-11 points. Episodic memory is evaluated by immediate and delayed recall of 10 Chinese words, with a score range of 0-10 points. If a participant’s score in either intelligence level or episodic memory is below one standard deviation for this dimension, they are assigned a score of 0; otherwise, they receive a score of 1. The cognitive dimension score is the sum of these two scores, ranging from 0-2 .

**Sensory dimension:** The sensory dimension is divided into two parts: vision and hearing. The vision section includes two questions: "How would you rate your eyesight for seeing things at a distance—excellent, very good, good, fair, or poor?" and "How would you rate your eyesight for seeing things up close—excellent, very good, good, fair, or poor?" Participants who rated their eyesight as excellent, very good, or good were given a score of 1, those who rated it fair received a score of 0.5, and those who rated it poor or were wearing glasses or blind were given a score of 0. The hearing section included the question, "How would you rate your hearing—excellent, very good, good, fair, or poor?" The scoring method for hearing was identical to that used for vision. The score of sensory dimension was the sum of the score of vision and hearing, ranging from 0-2.

**Vitality:** The vitality dimension is composed of two aspects: lung capacity and grip strength. When measuring lung capacity, participants stood upright, took a deep breath, placed their mouth around the mouthpiece, and exhaled with maximum force and speed. The peak expiratory flow rate was recorded. This measurement was repeated three times, with 30-second intervals between each attempt. The highest of the three peak expiratory flow rates was taken, and participants were scored based on threshold values of 350 L/min for males and 220 L/min for females, with scores of 0 or 1 assigned accordingly. For the grip strength measurement, participants stood with their elbow bent at a 90-degree angle by their side. They were instructed to squeeze the handgrip dynamometer with maximum force, hold for a few seconds, and then release. Each hand was measured twice, alternating between hands. The highest recorded value was used, with scores assigned based on threshold values of 28 kg for males and 18 kg for females, resulting in a score of either 0 or 1. The vitality dimension score is the sum of the lung capacity score and grip strength score, with a total range of 0-2.

**Psychological:** The 10-item version of the Center for Epidemiologic Studies Depression Scale (CESD-10) includes 10 items to assess depressive symptoms. Each item offers four response options based on the past week: little to no time (< 1 day), a small amount (1-2 days), sometimes or about half the time (3-4 days), and most or all of the time (5-7 days). The total score ranges from 0-30, with higher scores reflecting greater depressive symptoms. The psychological dimension was scored as follows: 0 for CESD scores 20-30, 1 for scores from 10-20, and 2 for scores from 0-10.

| **Variables** | **CHARLS validity** |
| --- | --- |
| Locomotion | qg002、qg003、qd002、qe002、qf002、qh002、qh003 |
| Sensory | da032、da033、da034、da035、da036、da037、da038、da039 |
| Vitality | qb002、qb003、qb004、qc003、qc004、qc005、qc006 |
| Psychological | dc009、dc010、dc011、dc012、dc013、dc014、dc015、dc016、dc017、dc018 |
| Cognitive | dc006、dc019、dc020、dc021、dc022、dc023、dc024、dc025、dc026、dc027 |

**Supplementary material of Mplus code**

DATA: FILE = DATA.DAT;

VARIABLE: NAME = ID P1-P10 lung grip walk

bala chair eye hear exe mem;

USEVARIABLE = P1-P10 lung grip walk

bala chair eye hear exe mem;

ANALYSIS: ESTIMATOR = ML;

MODEL: cogn BY mem exe;

loco BY walk bala chair;

vitality BY lung grip;

sensory BY eye hear;

psycho BY P1-P10;

IC BY mem* exe walk bala chair

lung grip eye hear P1-P10;

IC@1;

IC WITH cogn@0 loco@0 vitality@0 sensory@0 psycho@0;

OUTPUT: STAND CINT MOD;

# 2.Evaluation methods of health-related covariates.

Pain was assessed based on two self-reports of pain: “Are you often bothered by body pain?” and “Where in your body do you feel pain? Please list all parts of your body where you currently feel pain,” and the number of pain sites was categorized as no pain (0), single-site pain (1), and multisite pain (≥2). Chronic diseases were categorized by “Has a doctor ever told you that you have any of these chronic diseases?” obtained, including diseases such as hypertension, dyslipidemia, diabetes mellitus, and tumors, which were further classified as none (0), single (1), and multimorbidity (≥2) based on the type of chronic disease. Falls were categorized as no or yes by the item “Have you had a fall in the last two years”.

# 3.Supplementary material of Table S1

Supplementary Table S1 presents a comparison of baseline characteristics between participants with complete and incomplete data. Participants with incomplete data were significantly older (median age 68 vs. 66 years, *p* < 0.001) and had lower levels of education (*p* < 0.001) compared to those with complete data. No significant differences were observed in gender, economic level, pain status, chronic disease, or other characteristics.

**Table S1. Comparison of characteristics between participants with complete and incomplete data**

| **Variables** | **Complete data (N=4495)** | **Incomplete data (N=2087)** | ***p*** |
| --- | --- | --- | --- |
| Age, years, Median (IQR) | 66.00(62.00-71.00) | 68.00(63.00-75.00) | ＜0.001 |
| Gender N (%): Female | 2182(48.5) | 1014(48.6) | 0.974 |
| Male | 2313(51.5) | 1073(51.4) |  |
| Education N (%): Illiteracy | 2417(53.8) | 1196(57.3) | ＜0.001 |
| Primary school | 1190(26.5) | 440(21.1) |  |
| Middle School | 588(13.1) | 246(11.8) |  |
| High school and above | 300(6.7) | 199(9.5) |  |
| Missing | - | 6(0.3) |  |
| Economic level N (%): Low | 1733(38.6) | 736(35.3) | 0.765 |
| Medium | 1418(31.5) | 580(27.8) |  |
| High | 1344(29.9) | 577(27.6) |  |
| Missing | - | 194(9.3) |  |
| Pain N (%): No pain | 2936(65.3) | 1281(61.4) | 0.057 |
| Single-site pain | 317(7.1) | 163(7.8) |  |
| Multisite pain | 1242(27.6) | 612(29.3) |  |
| Miss | - | 31(1.5) |  |
| Chronic disease N (%): None | 677(15.1) | 262(12.5) | 0.273 |
| Singe | 1366(30.4) | 546(26.2) |  |
| Multimorbidity | 2452(54.5) | 1058(50.7) |  |
| Missing | - | 221(10.6) |  |
| Fall in the past 2 years N (%): Yes | 849(18.9) | 417(20.0) | 0.279 |
| No | 3646(81.1) | 1666(79.8) |  |
|  | - | 4(0.2) |  |
| Social Isolation(scores): 0 | 1392(31.0) | 541(25.9) | 0.096 |
| 1-2 | 2800(62.3) | 1142(54.7) |  |
| 3-4 | 303(6.7) | 150(7.2) |  |
| Missing | - | 254(12.2) |  |
| Health Lifestyle(scores): 0-2 | 1280(28.5) | 522(25.0) | 0.087 |
| 3 | 1661(37) | 610(29.2) |  |
| 4-5 | 1554(34.6) | 542(26.0) |  |
| Missing | - | 413(19.8) |  |

Note: IQR=interquartile range; IC=intrinsic capacity

# 4.Supplementary material of Table S2

Supplementary Table S2 shows the baseline characteristics of older participants categorized by social isolation and healthy lifestyle scores. Across social isolation groups, participants with higher scores (3-4) were older (median age 69 vs. 65 years), had a higher proportion of females (62.0% vs. 43.3%), and a greater percentage of illiteracy (71.3% vs. 43.2%) compared to those with a score of 0 (*p* < 0.001). Differences were also observed in economic level, pain status, and chronic disease prevalence (p< 0.05). No significant difference was found in fall history (*p* = 0.823).

Across healthy lifestyle groups, participants with higher scores (4-5) were younger (median age 65 vs. 66 years), more likely to be female (58.4% vs. 35.6%), and had lower rates of multisite pain and multimorbidity compared to those with lower scores (*p* < 0.05). Significant differences were also observed in education level, economic level, and fall history among the groups (*p* < 0.05).

**Table S2.** **Characteristics of the Older participants b****y Social Isolation, Health Lifestyle at Baseline**

| **Characteristic** | **Social Isolation(scores)** | | |  | **Health Lifestyle(scores)** | | |
| --- | --- | --- | --- | --- | --- | --- | --- |
|  | **0** | **1-2** | **3-4** |  | **0-2** | **3** | **4-5** |
| Age, years, Median (IQR) | 65.00(62.00-70.00) | 66.00(62.00-71.00) | 69.00(65.00-75.00) |  | 66.00(63.00-71.00) | 66.00(62.00-71.00) | 65.00(62.00-70.00) |
| *p* | ＜0.001 | | |  | ＜0.001 | | |
| Gender N (%): Female | 603(43.3) | 1391(49.7) | 188(62.0) |  | 456(35.6) | 819(49.3) | 907(58.4) |
| Male | 789(56.7) | 1409(50.3) | 115(38.0) |  | 824(64.4) | 842(50.7) | 647(41.6) |
| *p* | ＜0.001 | | |  | ＜0.001 | | |
| Education N (%): Illiteracy | 602(43.2) | 1599(57.1) | 216(71.3) |  | 640(50.0) | 873(52.6) | 904(58.2) |
| Primary school | 392(28.2) | 742(26.5) | 56(18.5) |  | 369(28.8) | 433(26.1) | 388(25.0) |
| Middle School | 233(16.7) | 332(11.9) | 23(7.6) |  | 186(14.5) | 244(14.7) | 158(10.2) |
| High school and above | 165(11.9) | 127(4.5) | 8(2.6) |  | 85(6.6) | 111(6.7) | 104(6.7) |
| *p* | ＜0.001 | | |  |  | ＜0.001 |  |
| Economic level N (%): Low | 414(29.7) | 1176(42.0) | 143(47.2) |  | 445(34.8) | 639(38.5) | 649(41.8) |
| Medium | 440(31.6) | 903(32.3) | 75(24.8) |  | 404(31.6) | 508(30.6) | 506(32.6) |
| High | 538(38.6) | 721(25.8) | 85(28.1) |  | 431(33.7) | 514(30.9) | 399(25.7) |
| *p* | ＜0.001 | | |  | ＜0.001 | | |
| Pain N (%): No pain | 999(71.8) | 1748(62.4) | 189(62.4) |  | 796(62.2) | 1076(64.8) | 1064(68.5) |
| Single-site pain | 91(6.5) | 201(7.2) | 25(8.3) |  | 95(7.4) | 113(6.8) | 109(7.0) |
| Multisite pain | 302(21.7) | 851(30.4) | 89(29.4) |  | 389(30.4) | 472(28.4) | 381(24.5) |
| *p* | ＜0.001 | | |  | 0.001 | | |
| Chronic disease N (%): None | 216(15.5) | 421(15) | 40(13.2) |  | 172(13.4) | 236(14.2) | 269(17.3) |
| Singe | 384(27.6) | 898(32.1) | 84(27.7) |  | 368(28.7) | 479(28.8) | 519(33.4) |
| Multimorbidity | 792(56.9) | 1481(52.9) | 179(59.1) |  | 740(57.8) | 946(57.0) | 766(49.3) |
| *p* | 0.019 | | |  | ＜0.001 | | |
| Fall in the past 2 years N (%): Yes | 265(19) | 528(18.9) | 56(18.5) |  | 266(20.8) | 326(19.6) | 257(16.5) |
| No | 1127(81) | 2272(81.1) | 247(81.5) |  | 1014(79.2) | 1335(80.4) | 1297(83.5) |
| *p* | 0.823 | | |  | 0.003 | | |

Note: IQR=interquartile range

# 5. Supplementary material of Table S3

Table S3 reported the results about the cross-sectional associations between social isolation, healthy lifestyle, and IC at baseline. Compared to participants with no social isolation (score = 0), those with moderate (1-2) and high (3-4) levels of social isolation had significantly lower IC scores (B = −0.39 and −0.60, respectively; *p* < 0.001). In contrast, healthier lifestyle scores were positively associated with IC. Participants scoring 3 and 4-5 had significantly higher IC scores compared to those with scores 0-2 (B = 0.35 and 0.58, respectively; *p* < 0.001). Among covariates, older age, female gender, lower education, lower economic status, presence of pain, multimorbidity, and history of falls were significantly associated with lower IC (*p* < 0.001). Multisite pain (B = -0.98) and increasing age (B = −0.06 per year) showed the strongest negative associations with IC.

**Table S3. Cross-sectional Association of Social Isolation and** **Health Lifestyle with IC among Older Adults**

| **Variables** | **B(95%CI)** | ***SE*** | ***β*** | ***t*** | ***p*** | ***VIF*** |
| --- | --- | --- | --- | --- | --- | --- |
| Constant | 10.67(10.14-11.21) | 0.271 |  | 39.416 | ＜0.001 |  |
| Social Isolation(scores) (ref: 0) |  |  |  |  |  |  |
| 1-2 | -0.39(-0.49--0.30) | 0.05 | -0.109 | -7.939 | ＜0.001 | 1.191 |
| 3-4 | -0.60(-0.79--0.42) | 0.096 | -0.086 | -6.289 | ＜0.001 | 1.191 |
| Health Lifestyle(scores) (ref: 0-2) |  |  |  |  |  |  |
| 3 | 0.35(0.25-0.46) | 0.055 | 0.097 | 6.386 | ＜0.001 | 1.474 |
| 4-5 | 0.58(0.47-0.69) | 0.057 | 0.156 | 10.07 | ＜0.001 | 1.535 |
| Age | -0.06(-0.07--0.05) | 0.004 | -0.208 | -16.19 | ＜0.001 | 1.049 |
| Gender (ref: Female) |  |  |  |  |  |  |
| Male | 0.31(0.22-0.41) | 0.048 | 0.088 | 6.472 | ＜0.001 | 1.187 |
| Education (ref: Illiteracy) |  |  |  |  |  |  |
| Primary school | 0.66(0.55-0.77) | 0.055 | 0.165 | 11.972 | ＜0.001 | 1.209 |
| Middle School | 0.86(0.72-1.00) | 0.072 | 0.165 | 12.021 | ＜0.001 | 1.207 |
| High school and above | 0.78(0.59-0.97) | 0.097 | 0.111 | 8.069 | ＜0.001 | 1.203 |
| Economic level (ref: Low): |  |  |  |  |  |  |
| Medium | 0.17(0.07-0.28) | 0.053 | 0.045 | 3.195 | 0.001 | 1.262 |
| High | 0.34(0.23-0.45) | 0.057 | 0.089 | 6.022 | ＜0.001 | 1.383 |
| Pain (ref: no pain) |  |  |  |  |  |  |
| Single-site pain | -0.49(-0.66--0.31) | 0.088 | -0.071 | -5.536 | ＜0.001 | 1.039 |
| Multisite pain | -0.98(-1.08--0.88) | 0.053 | -0.249 | -18.463 | ＜0.001 | 1.161 |
| Chronic disease (ref: none ) |  |  |  |  |  |  |
| Singe | 0.01(-0.13-0.15) | 0.07 | 0.002 | 0.12 | 0.905 | 2.111 |
| Multimorbidity | -0.27(-0.40--0.15) | 0.066 | -0.077 | -4.166 | ＜0.001 | 2.197 |
| Fall in the past 2 years (ref: no) |  |  |  |  |  |  |
| Yes | -0.44(-0.56--0.33) | 0.057 | -0.099 | -7.719 | ＜0.001 | 1.038 |

Note: SE=standard error; VIF=Variance Inflation Factor

# 6. A detailed description of the results of Model 1- Model 4 in Table 2

The results in Table 2 show the longitudinal relationship between social isolation, healthy lifestyle, and IC. Model 1, which incorporated only social isolation, showed a significant decrease in IC for participants with a social isolation score of 1-2 (β = −0.65, 95%CI: −0.71 to −0.58) and 3-4 (β = −1.30, 95%CI: −1.43 to −1.17) compared to no social isolation (score of 0) . Model 2, which included only healthy lifestyle, showed significantly higher IC for participants scoring 3 (β = 0.26, 95%CI: 0.18 to 0.34) and 4-5 (β = 0.58, 95%CI: 0.50 to 0.66) compared to those scoring 0-2. Model 3 incorporated both social isolation and healthy lifestyle, with participants with social isolation scores of 1-2 and 3-4 experiencing a decrease in IC (β = −0.65, 95%CI: −0.72 to −0.59) and (β = 1.31, 95%CI: −1.44 to −1.17), and those with healthy lifestyle scores of 3 and 4-5 experiencing an elevated IC (β = 0.27, 95%CI: 0.20 to 0.35) and (β = 0.59, 95%CI: 0.51 to 0.67), respectively. Model 4 adjusted for gender, age, and education, participants with social isolation scores of 1-2 and 3-4 had decreased IC of (β = −0.40, 95%CI: −0.46 to −0.33) and (β = −0.69, 95%CI: −0.81 to −0.57), respectively, and those with healthy lifestyle scores of 3 and 4-5 had elevated IC of (β = 0.33, 95%CI: 0.26 to 0.40) and (β = 0.68, 95%CI: 0.61 to 0.75), respectively.

# 7. A detailed description of the results of Model 1- Model 4 in Table 2

Supplementary Table S3 shows the longitudinal association of social Isolation and health lifestyle and IC dimensions. Social isolation was significantly associated with lower scores in most IC domains, particularly in cognition and psychological functioning. Compared with participants without social isolation (score of 0), those with moderate (1-2) and high (3-4) levels of social isolation showed lower cognitive function (β = −0.11, 95%CI: −0.14 to −0.09; β = −0.21, 95%CI: −0.26 to −0.16, respectively) and psychological capacity (β = −0.12, 95% CI: −0.14 to −0.10; β = −0.26, 95%CI: −0.30 to −0.21, respectively; *p* < 0.001). Similar but weaker associations were observed for locomotion and vitality, while associations with sensory function were not statistically significant.

A higher healthy lifestyle score was positively associated with locomotion and psychological domains. Participants with a lifestyle score of 4-5 had significantly better locomotor performance (β = 0.33, 95%CI: 0.30 to 0.37, *p*< 0.001) and psychological health (β = 0.09, 95%CI: 0.06 to 0.12, *p*< 0.001) compared to those with a score of 0-2. Associations with sensory and vitality domains were modest, while no significant associations were found for cognition.

**Table S4. Longitudinal association of Social Isolation and Health Lifestyle and IC dimensions**

| **Characteristics** | **Model 1** | **Model 2** | **Model 3** | **Model 4** | **Model 5** |
| --- | --- | --- | --- | --- | --- |
|  | **Locomotion**  β(95%CI) p-value | **Cognition**  β(95%CI) p-value | **Sensory**  β(95%CI) p-value | **Vitality**  β(95%CI) p-value | **Psychological**  β(95%CI) p-value |
| **Social Isolation(scores) (ref: 0)** | | | | | |
| **1-2** | -0.08  (-0.11- -0.05)*** | -0.11  (-0.14- -0.09)*** | -0.02  (-0.04- 0.00) | -0.05  (-0.08- -0.02)*** | -0.12  (-0.14- -0.10)*** |
| **3-4** | -0.10  (-0.16- -0.04)*** | -0.21  (-0.26- -0.16)*** | -0.03  (-0.08- 0.01) | -0.04  (-0.09- -0.02)* | -0.26  (-0.30- -0.21)*** |
| **Health Lifestyle(scores)(ref: 0-2)** | | | | | |
| **3** | 0.18  (0.15- 0.21)*** | 0.01  (-0.01- 0.04) | 0.02  (-0.01- 0.04) | -0.04  (-0.09- 0.02) | 0.06  (0.03- 0.07)*** |
| **4-5** | 0.33  (0.30- 0.37)*** | 0.01  (-0.02- 0.04) | 0.05  (0.02- 0.08)*** | 0.04  (0.04- 0.05)* | 0.09  (0.06- 0.12)*** |
| **AIC** | 22868.436 | 19905.637 | 17069.207 | 20267.863 | 17182.919 |
| **BIC** | 22882.942 | 19920.143 | 17083.713 | 20286.370 | 17197.425 |

Note: Boldface indicates statistical significance (**p*<0.05, *** *p*<0.001)

All Models were adjusted for age, gender, education, economic level, pain, fall down, and chronic disease
